# A comparison of *Plasmodium falciparum* circumsporozoite protein-based slot blot and ELISA immuno-assays for oocyst detection in mosquito homogenates

**DOI:** 10.1186/s12936-015-0954-2

**Published:** 2015-11-14

**Authors:** Will Stone, Bryan Grabias, Kjerstin Lanke, Hong Zheng, Emily Locke, Diadier Diallo, Ashley Birkett, Merribeth Morin, Teun Bousema, Sanjai Kumar

**Affiliations:** Department of Medical Microbiology, Radboud University Nijmegen Medical Centre, Nijmegen, The Netherlands; Laboratory of Emerging Pathogens, Division of Emerging and Transfusion Transmitted Diseases, Office of Blood Research and Review, Center for Biologics Evaluation and Research, Food and Drug Administration, Rockville, MD USA; PATH Malaria Vaccine Initiative, Washington DC, USA; Department of Immunology and Infection, London School of Hygiene and Tropical Medicine, Keppel Street, London, UK

**Keywords:** *Plasmodium falciparum*, *Anopheles*, Sporozoite, Oocyst, Circumsporozoite protein, ELISA, Slot-blot, Immuno-assay, Mosquito infection

## Abstract

**Background:**

The infectivity of *Plasmodium* gametocytes is typically determined by microscopically examining the midguts of mosquitoes that have taken a blood meal containing potentially infectious parasites. Such assessments are required for the development and evaluation of transmission-reducing interventions (TRI), but are limited by subjectivity, technical complexity and throughput. The detection of circumsporozoite protein (CSP) by enzyme-linked immunosorbent assay (ELISA) and enhanced chemiluminescent slot-blot (ECL-SB) may be used as objective, scalable alternatives to microscopy for the determination of infection prevalence.

**Methods:**

To compare the performance of the CSP ELISA and ECL-SB for the detection of mosquito infection, four groups of *Anopheles stephensi* mosquitoes were infected with cultured *Plasmodium falciparum* gametocytes. At day-8 post-infection (PI), parasite status was determined by microscopy for a sample of mosquitoes from each group. At days 8 and 10 PI, the parasite status of separate mosquito samples was analysed by both CSP ELISA and ECL-SB.

**Results:**

When mosquito samples were analysed 8 days PI, the ECL-SB determined similar infection prevalence to microscopy; CSP ELISA lacked the sensitivity to detect CSP in all infected mosquitoes at this early time point. When mosquitoes were analysed 48 h later (10 days PI) both assays performed as well as microscopy for infection detection.

**Conclusions:**

Whilst microscopical examination of mosquito guts is of great value when quantification of parasite burden is required, ECL-SB and CSP ELISA are suitable alternatives at day 10 PI when infection prevalence is the desired endpoint, although CSP ELISA is not suitable at day 8 PI. These results are important to groups considering large-scale implementation of TRI.

**Electronic supplementary material:**

The online version of this article (doi:10.1186/s12936-015-0954-2) contains supplementary material, which is available to authorized users.

## Background

Transmission-reducing interventions (TRIs) that specifically aim to interrupt the transmission of malaria from man to mosquito form important components of malaria control and elimination strategies [1, 2]. The development, targeting and evaluation of TRIs requires robust assessments of the infectivity of human malaria infections to mosquitoes [3, 4]. Mosquito infection status is typically determined by microscopy following the observation of *Plasmodium* oocysts in the midgut or sporozoites in the salivary glands [5, 6]. Oocysts develop on the basal lamina of the mosquito midgut approximately 2 days after the ingestion of a blood meal containing infectious gametocytes, and can be visually detected by microscopy approximately 6 days post infection (PI) [5]. Sporozoites develop from the budding of sporoblasts in the developing oocyst, which give rise to hundreds or thousands of sporozoites in an explosive population expansion that starts 7–8 days PI [7, 8]. Beyond 10 days PI, sporozoites rupture the oocyst capsule and enter the haemolymph, beginning their migration to the mosquito salivary glands [7, 9]. Within 8 h of their release, sporozoites must invade the salivary glands or else be broken down in the mosquito haemolymph [10]. Sporozoites that succeed in entering the salivary glands are detectable from about 11 days PI, and the number in the glands appears to plateau after approximately 14 days PI [11] where they remain viable for long periods [12, 13]. Because of this stability and because very few sporozoites are egested during blood feeding [14, 15] it is generally accepted that a mosquito with any number of salivary gland sporozoites is infective to humans.

The goal of TRIs is to reduce the proportion of mosquitoes becoming infectious after taking a blood meal on vaccinated or treated individuals [16]. A recent study showed that eventual infectivity can be predicted with reasonable certainty from the detection of maturing oocysts in low-intensity infections [11]. With highly trained technicians, mosquito dissections can be performed quickly; however, the ability to properly identify and quantify developing oocysts is a specialized skill that requires long periods of training. Furthermore, the necessity to screen individual midguts during a limited time-window PI limits the throughput of mosquito feeding assays. It is highly desirable that the endpoint for efficacy assessments of TRIs be unambiguous, flexible with regard to the timing of mosquito processing, and usable by non-specialized staff. Screening mosquitoes for oocyst stage infections by high throughput immunological or molecular tools may provide an alternative to microscopy for processing large volumes of mosquitoes.

Circumsporozoite protein (CSP) is a ~60 kD glycosylphosphatidyl-inositol (GPI)-anchored sporozoite, surface-coat protein with roles in parasite development in oocysts [17], traversal of the haemocoel [18], recognition and binding to the salivary glands [19, 20], protection after egestion into the human microvasculature [18], and invasion of human hepatocytes [21, 22]. In the mosquito, CSP is abundantly expressed by the developing parasite [23], making the protein an ideal target for immuno-assays. The colorimetric enzyme-linked immunosorbent assay (ELISA) is commonly used for the detection of *Plasmodium* CSP in wild-caught mosquitoes [6, 24–26], where salivary gland infection (measured by homogenization of the head or head and thorax) has generally been the endpoint of interest. A positive ELISA test result indicates that mosquitoes ingested infectious parasites more than 11 days prior to assessment. If the ELISA is performed at 7 days PI, when oocysts are visible by microscopy, CSP expression by developing oocysts is too low for detection [11]. The enhanced chemi-luminescent ELISA (ECL ELISA) and slot-blot assays (ECL-SB) were designed to overcome these issues of sensitivity, and are capable of detecting as little as 4.4 and 1 pg of recombinant CSP, respectively [27, 28], which could allow for *Plasmodium* detection shortly after the onset of CSP production during oocyst development.

Here, two immunoassays, the CSP ELISA and ECL-SB, were evaluated for their ability to detect CSP in mosquito homogenates processed from whole mosquito carcasses, by comparison to mosquito samples analysed by standard dissection and oocyst enumeration. Gametocyte cultures were diluted to ensure parasite burden in test mosquitoes was close to the level of *Plasmodium*-infected vectors from endemic regions [29, 30], providing groups of mosquitoes with controlled, but varying infection prevalence to investigate the performance of the two assays relative to microscopy for the detection of oocyst-stage infections. Since CSP expression increases during oocyst maturation [27, 31], the sensitivity of the assays in longer term infected mosquito specimens that contain a higher amount of CSP was also examined.

## Methods

### Infection of *Anopheles stephensi* mosquitoes with *Plasmodium falciparum* parasites

1. *Mosquito rearing*: *Anopheles stephensi* (Sind-Kasur Nijmegen strain) [32] were reared at 30 °C and 70–80 % humidity, while exposed to a 12/12 h day/night cycle.
2. *Parasite culture*: Mature *Plasmodium falciparum* (NF54) gametocytes (14-day culture, 0.3–0.5 % gametocytes, 2 % haematocrit) were obtained from an automated tipper system and prepared as previously described [33, 34]. To achieve low-intensity infections, infective blood meals that are routinely used and produce high infection prevalence (averaging >70 % with mean oocyst intensities in infected mosquitoes >10) were diluted at a ratio of 1:10 with uninfected blood.
3. *Mosquito feeding assays*: Four separate batches of gametocyte material were each fed to multiple cages of mosquitoes (10 cages total). For each cage, approximately 150 three- to five-days old *Anopheles stephensi* mosquitoes were fed on a glass membrane midi-feeder system containing ~1.25 mL of the *P. falciparum* culture mix [33, 34]. Unfed and partially fed mosquitoes were removed after feeding and blood-fed females were maintained at 26 °C and 70–80 % humidity.
4. *Experimental design*: After infection, mosquitoes were combined to have four large batches of mosquitoes that allowed examination by microscopy, CSP ELISA and ECL-SB. At day 8 PI, 20–30 mosquitoes per batch were examined for oocysts by microscopy. At days 8 and 10 PI, 36–48 mosquitoes per batch were stored at −20 °C in sealed containers until analysis by CSP ELISA and ECL-SB. Sample sizes for the two assays were maximised based on the availability of live mosquitoes at the two time points and were kept uniform between the two assays. Left-over mosquitoes were killed and discarded. Full details of mosquito sample sizes for all assays and groups are in Table 1.

**Table 1:** *Plasmodium falciparum* oocyst prevalence determined by microscopy (day 8 PI) and by the detection of CSP in the ECL-SB and colorimetric ELISA in separate mosquito samples processed 8 and 10 days PI from the same four experimental feeds

Group	Day PI	Microscopy				ECL-SB				ELISA			
		Mean oocysts (range)	Prevalence %	95 % CI	n/N	Prevalence %	95 % CI	n/N	p	Prevalence %	95 % CI	n/N	p
A	8	0.5 (2–4)	*20.8*	7.1–42.2	5/24	*27.8*	14.2–45.2	10/36	0.54	*0.0*	0–9.7	0/36	0.00
	10	–	*–*	–	–	*25.0*	12.1–42.2	9/36	0.71	*33.3*	18.6–51	12/36	0.29
B	8	0.9 (1–3)	*40.0*	19.1–63.9	8/20	*37.5*	24–52.6	18/48	0.85	*0.0*	0–7.4	0/48	0.00
	10	–	*–*	–	–	*39.6*	25.8–54.7	19/48	0.97	*43.8*	29.5–58.8	21/48	0.78
C	8	6.1 (1–22)	*70.0*	50.6–85.3	21/30	*75.0*	57.8–87.9	27/36	0.65	*11.1*	3.1–26.1	4/36	0.00
	10	–	*–*	–	–	*80.6*	64–91.8	29/36	0.32	*75.0*	57.8–87.9	27/36	0.65
D	8	7.7 (1–17)	*93.3*	77.9–99.2	28/30	*91.7*	80–97.7	44/48	0.79	*14.6*	6.1–27.8	7/48	0.00
	10	–	*–*	–	–	*87.5*	74.8–95.3	42/48	0.41	*87.5*	74.8–95.3	42/48	0.41

*Group* group of mosquitoes fed on the same gametocyte culture, *Mean oocysts* (*range*) mean oocysts is given as the mean of all mosquitoes sampled. Range is given as the range of oocyst numbers in positive infections, *Day PI* day post infection, *n/N* positive mosquitoes/total mosquito sample size, *P* Chi squared test *p* value for homogeneity between positivity rates in the ECL-SB and ELISA relative to microscopy

### Microscopy

Routine staining of midguts for oocyst detection was done in 1 % mercurochrome, as described previously [11, 33]. All oocyst detection was performed once by expert staff at Radboud UMC, Nijmegen, The Netherlands.

### ECL slot-blot

**Fig. 1:**
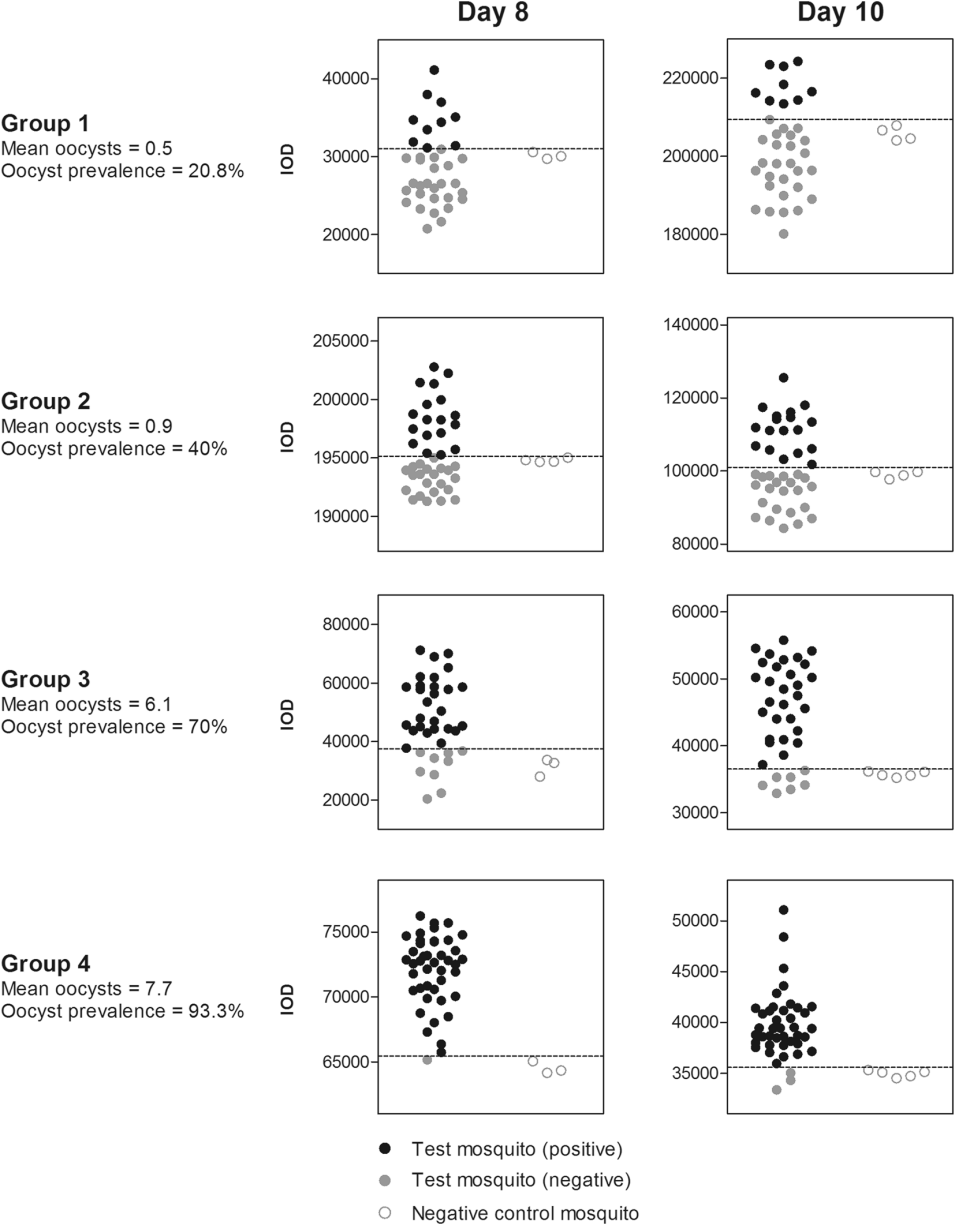
ECL-SB integrated optical density values for mosquito homogenate bands exposed by X-ray. The capacity of the slot blot apparatus was 48 mosquito homogenates, including three to five negative controls for the calculation of CSP positivity. *Each blot* was developed separately, giving rise to the varied IOD values in the figure. Positivity determination was thus blot-specific, based on the mean IOD values of the negative controls obtained in *each blot*. As the total number of mosquitoes processed at each time point in groups 2 and 4 exceeded the capacity of a single blot (48 test mosquitoes plus controls), a small number of test mosquitoes from these groups were processed in separate blots (eight and five mosquitoes from each time point for groups 2 and 4, respectively), the results of which are shown in Additional file 1: Figure S1. For clarity, the primary *figure* shows *blots* containing the majority of mosquitoes from all groups and time points

1. *Preparation of whole mosquito homogenates*: CSP expression on the developing oocysts was determined in lysed whole mosquitoes using a procedure described previously [27]. Briefly, individual blood-fed or unfed mosquitoes were placed into single tubes and homogenized with a piston in 50 μl of lysis buffer (1× TBS, 0.5 % SDS). The lysates were subsequently vortexed for 20 s and then boiled for 5 min. The insoluble material was pelleted via centrifugation and the supernatant was collected and analysed.
2. *mAb 2A10*: Anti-Pf CSP mAb 2A10 was generated using a hybridoma cell line acquired from the MR4/ATCC, Virginia, USA. A commercial source was used to produce ascites in mice and purify antibodies by Protein G affinity chromatography (Harlan Laboratories Inc. Madison, WI, USA). mAb 2A10 (1.55 mg/mL) immune-reactivity was characterized in IFA using *P. falciparum* sporozoites and in ELISA and Western Blot using rPf CSP.
3. *Performance of the ECL-SB*: This assay was performed using a Minifold 48 slots, Whatman apparatus (GE Healthcare Life Sciences, 10447941; Piscataway, NJ, USA) in a slightly modified version of the standard protocol [27]. The ECL-reagents used in this assay were purchased as a kit (Life Technologies, Western-StarTM Immunodetection System, T1046, Grand Island, NY, USA). Approximately 20 µL of each sample lysate was loaded into each slot-blot well. Sample proteins were allowed to adsorb onto the nitrocellulose membrane for 1 h and then slots were washed with 500 µL of deionized water four times. The membrane was next blocked at room temperature (RT) in iBlock blocking buffer (Applied Biosystems, T2015, Foster City, CA, USA) for 1 h before being probed with anti-Pf CSP mAb 2A10 (at 0.31 µg/mL) for an additional hour. The membrane was subsequently washed three times (5 min each) in deionized water and three times in iBlock blocking buffer. After the washings, the membrane was incubated with an AP-conjugated ECL-goat anti-mouse-IgM + IgG secondary antibody (1:5000 dilution) for 1 h at RT and then washed again as described following incubation with mAb 2A10. Before development, the membrane was rinsed twice for 2 min with 25 mL of 1× assay buffer and bands were visualized by incubating the membrane in 6 mL of ECL-substrate solution at RT for 5 min and exposure to an AR film (Kodak X-OMAT 1000A).
4. *ECL slot blot data acquisition and analysis*: The band profile on the developed film was scanned and analysed using the ImageJ program [35]. The integrated optical density (IOD) of each band was determined by measuring the band intensity in a ‘gated area’. The dimensions of the gated area for IOD determination was kept constant for each band on the scanned image. The capacity of a single blot was 48 mosquito samples, including three to five negative controls for the calculation of CSP positivity. Each blot was developed separately. As there were large variations in IOD values between blots (Fig. 1), positivity determination for test mosquitoes was blot-specific, based on the mean IOD values of the negative controls processed in each blot. As the total number of mosquitoes processed at each time point in groups 2 and 4 exceeded the capacity of a single blot (48 test mosquitoes plus controls), a small number of test mosquitoes from these groups were processed in separate blots (8 and 5 mosquitoes from each time point for groups 2 and 4, respectively), the results of which are shown in Additional file 1: Figure S1. Cut-off values for the determination of positive and negative specimens in each run of the experiment were determined as the mean plus two times the standard deviation of the uninfected mosquito band intensities, as described previously [27].

### ELISA

1. *3SP2 mAb*: Anti-Pf CSP mAb 3SP2 was generated at Radboud UMC, Nijmegen, The Netherlands as described previously [14, 26].
2. *CSP-ELISA*: CSP ELISA was performed as previously described [11]. Mosquitoes were homogenized in 250 μl phosphate buffered saline (PBS pH 7.2) solution with 1 % sarcosil. Sterilin ELISA plates were coated with 3SP2 mAb at 5 µg/mL, diluted in PBS. Fifty microliters of mosquito homogenate from all test mosquitoes was analysed in duplicate (100 μl total), alongside blank wells (50 μl of sample diluent) and a standard curve of recombinant CSP (50 µL Gennova, 0.1 µg/mL). A selection of homogenates from 15 uninfected blood-fed control mosquitoes were tested on every plate as a visual control, giving OD values between 0.06 and 0.08. OD values were adjusted for plate-to-plate variation by subtracting plate blank values. A universal cut-off for CSP positivity was determined at an optical density (OD) of 0.311 using maximum likelihood methods to establish CSP-negative and CSP-positive Gaussian distributions from the corrected OD values of all 336 test mosquitoes. The cut-off was set as the mean OD of the CSP-negative distribution plus three standard deviations, as previously described [36, 37].

### Statistical analysis

Statistical significance between ECL Slot-blot and ELISA-based prevalence estimates and those measured by microscopic detection after dissection of mosquito midguts were evaluated using the Chi square test for homogeneity. Statistical analysis was conducted in GraphPad Prism 5.0 (GraphPad Software Inc, CA, USA), and confidence intervals for prevalence estimates were generated using STATA 12 (StataCorp., TX, USA).

## Results

### Infection of *Anopheles stephensi* mosquitoes with *Plasmodium falciparum* gametocyte cultures

Mosquitoes were fed blood meals containing four batches of gametocytes from *P. falciparum* cultures diluted to generate varying infection rates (Table 1). Parasite burdens and overall infection prevalences were estimated after mosquito dissection and oocyst detection and counting by microscopy on a subset of 20–30 randomly sampled mosquitoes from each group on day 8 PI. Additional paired samples of 36–48 mosquitoes were then removed from each of the four groups on days 8 and 10 PI for processing and assessment in the ECL-SB and ELISA (one sample for each day and method). The mosquitoes were randomly selected from the cages fed on the same gametocyte material by the same feeders. Since different mosquitoes were used for each of the three assays, some inherent variation in mosquito infection rates was expected. Full details of sample sizes in different test groups for all assays are in Table 1. In total, 104 test mosquitoes were analysed by microscopy on day 8 PI, 336 test mosquitoes were analysed in the ECL-SB on days 8 and 10 PI, and 336 test mosquitoes were analysed in the ELISA on days 8 and 10 PI.

### Oocyst detection by microscopy

Mean oocyst intensities for groups 1 through 4 were 0.5, 0.9, 6.1 and 7.7 (range 0–22), with corresponding oocyst prevalences of 20.8, 40, 70, and 93.3 %, respectively, when assessed on day 8 PI (Table 1). The percentages of infected mosquitoes with only a single oocyst were 0, 25, 11 and 5 %, respectively for groups 1 through 4.

### Prevalence estimation using ECL-SB

Table 1 shows that at all oocyst intensities and time points the ECL-SB accurately estimated oocyst prevalence as detected by microscopy in mosquito samples from the same feed. All prevalence estimates were made using a cut-off threshold of two standard deviations from the mean of the IOD (integrated optical density) values obtained from ECL-SB from uninfected mosquitoes, as described previously [27]. Actual IOD values vary significantly between separate assay repeats according to the relative time exposed to X-ray, but positive/negative determination was consistently robust within experiments (Fig. 1).

### Prevalence estimation using ELISA

**Fig. 2:**
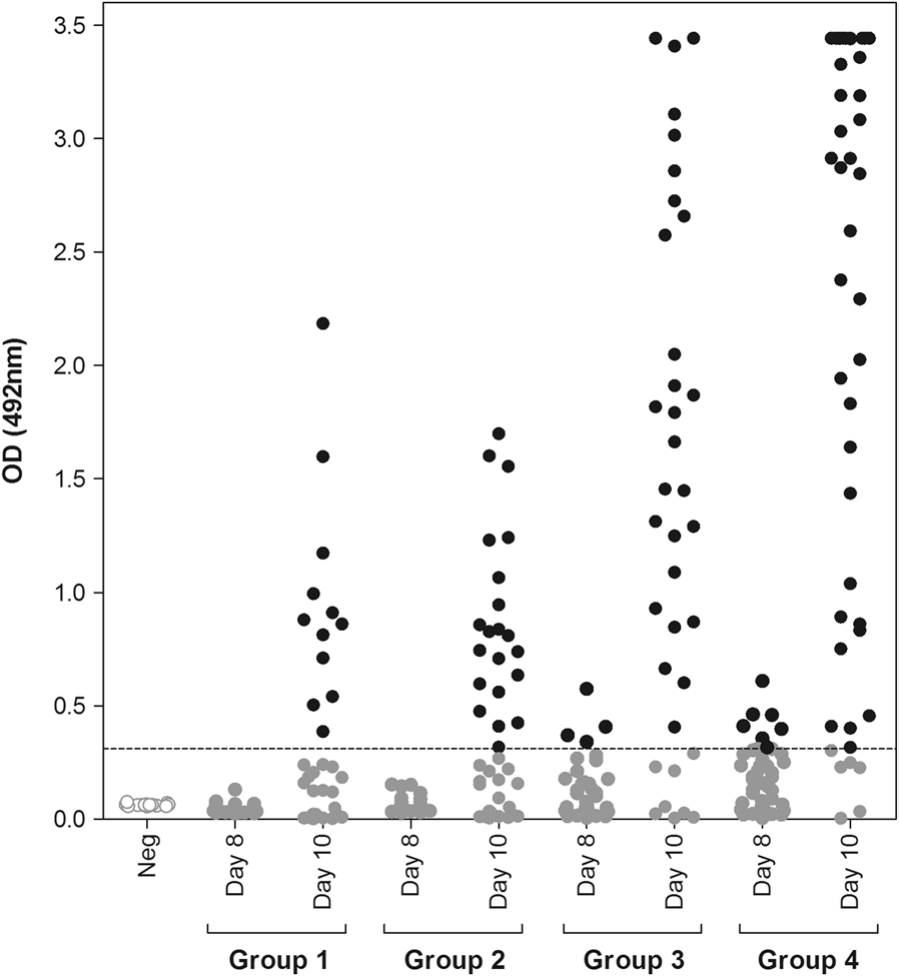
ELISA OD values for all test and negative control mosquitoes. Plate-specific correction of OD values against background reactivity allowed for the calculation of a universal cut-off for positivity, which was determined statistically (OD 0.311, shown as the *dotted line*) [36, 37, 43]. *Data points* are colored as in Fig. 1

In line with previous results [11], the ELISA was not sufficiently sensitive to detect CSP present in infected mosquitoes at day 8 PI, regardless of oocyst intensity (Fig. 2). However, after an additional 48 h, the ELISA accurately estimated the microscopically determined oocyst prevalence at all oocyst intensities. No significant differences were observed between prevalence estimates made by ELISA and microscopy (Table 1; p > 0.29), or between ELISA and ECL-SB compared directly (p ≥ 0.4) for mosquitoes analysed at day 10 PI.

## Discussion

This study describes the ability of two immuno-assays, the CSP-ELISA and ECL-SB, to detect low-density mosquito-stage *P. falciparum* infections based on the detection of CSP in the mosquito carcass. The results suggest that when mosquitoes are assayed 10 days after ingestion of an infective blood meal containing gametocytes, infection detection by the CSP-ELISA and ECL-SB provide prevalence estimates that are comparable to those obtained by microscopy. If mosquitoes are assayed earlier (8 days PI), only the ECL-SB has the sensitivity to accurately estimate true infection prevalence, as compared to microscopy.

The results of the current study corroborate recent data indicating that the ECL-SB can detect CSP shortly after its production begins in parallel to the budding out of sporozoites in midgut oocysts [31]. Marginally higher prevalence estimates at day 10 PI compared to day 8 PI in groups 2 and 3, which contained single oocyst infections (mean oocyst intensity 0.9–6.1) suggest that some slower developing oocysts in low-intensity infections may be missed if the assay is performed early. The challenges to detecting developing oocysts on day 8 PI was more pronounced when the colorimetric ELISA was used [11]. A third method, the ECL-ELISA, was recently proposed as an enhancement of the standard colorimetric ELISA for the detection of CSP in whole homogenized mosquito samples [28]. This assay is capable of detecting an amount of CSP equivalent to that produced by 1.7 oocysts in mosquitoes processed at day 8 PI, a significant improvement on the colorimetric ELISA, although still possibly incapable of detecting single oocyst infections at this time point. Though the experiments were not powered to investigate the impact of oocyst intensity on assay sensitivity, the similar level of concordance with microscopy across the range of infection intensities we observed (1–22 oocysts) indicate that both ECL-SB and ELISA reliably detected single oocyst infections at day 10 PI; the ECL-SB also detecting the vast majority at day 8 PI. ECL-SB prevalence estimates were in fact most concordant with microscopy in mosquito groups with only 1–4 oocysts (1 and 2), including group 2 where 25 % of all infected mosquitoes sampled by microscopy harboured only a single oocyst. This indicates that the assays used would have great utility for the detection oocysts in *Plasmodium* infected vectors from endemic areas (e.g. mosquitoes infected during direct mosquito feeding assays), which are commonly in the 1–5 oocyst range [29, 38].

In the context of public health, the prevalence of mosquitoes that are infectious to humans is the most relevant output for determining the efficacy of TRIs [16, 39]. Oocysts may produce many thousands of sporozoites, the majority capable of invading and establishing themselves in the salivary glands where they await egestion by the blood-feeding mosquito into the human dermis [12, 13]. Since mosquitoes egest very few sporozoites when feeding, a mosquito with any number of sporozoites is probably infectious [14, 15].

While the presence of salivary gland sporozoites marks infectiousness to humans, detecting earlier developmental stages may have significant operational benefits. For mosquito-feeding experiments, storage time after feeding is a major concern as mosquito mortality may drastically limit sample size for analysis [11, 40, 41]. Early infection detection based on oocyst detection is reliable from day 6 PI by microscopy and, although a minority of oocysts may fail to produce sporozoites [9, 11], reliably predicts later sporozoite salivary gland infection [11]. Oocysts are thereby detectable before sporozoite proliferation, however, the routine microscopical detection of oocysts has a subjective element and requires highly trained microscopists to reliably detect low-density infections. CSP-based assays form an attractive alternative to microscopy because of CSP abundance, and specificity to the oocyst and sporozoite stages of sporogonic development [23]. Though CSP detection necessitates mosquito processing at day 8 PI or later [11, 27, 28], the fact of its detection may be a more reliable predictor of actual mosquito infectivity than the observation of oocyst capsules or presence of parasite DNA. For the assays in the current study, requirements for mosquito processing are modest, and equipment and assay methods relatively low-tech.

One element that is consistent for ELISA, ECL-SB and other immunological approaches to infection detection in whole mosquito homogenates is the method of mosquito homogenization. This has the benefit that mosquitoes may be killed in their cages by removal to −20 °C freezers, then moved into sealed storage until analysis. Microscopy is constrained by strict scheduling based on the dates of experimental infections, whereas immunological and molecular assays may be separated from the schedule of feeding experiments at the convenience of the operator.

Another similarity between the ELISA and the ECL-SB is the relative low cost and opportunities to increase throughput, which is beneficial for low-resource settings and for settings where the proportion of infected mosquitoes is low. For the assays performed in this study, the estimated cost per mosquito was 0.96 USD for the ECL-SB and <0.1 USD for CSP ELISA. For the ECL-SB as performed here, 48 mosquitoes per apparatus can be assayed each day. For the ELISA, throughput is even higher and technology and equipment are routinely available in most research laboratories. For the CSP ELISA as performed here, 96 mosquitoes can be assayed per plate and ten plates can easily be processed by a single technician, with assays being completed in a 2-day period.

## Conclusions

The results of this and previous studies indicate that either the ECL-SB or ELISA may replace microscopy for infection detection at day 10 PI, and that ECL-SB may do so 2 days earlier with no risk to assay sensitivity. Samples were not tested at day 9 PI, but it is possible the ELISA may have performed better at this time point. Availability of novel early midgut stage and trans-midgut stage, non-CSP based biomarkers may further improve the sensitivity of immunological assays for *Plasmodium* detection in mosquitoes. Efforts to develop such assays for trans-midgut stage (days 2–10 PI) *Plasmodium* detection are underway. The ECL-SB’s throughput is dependent on the number of apparatuses available, while the ELISA’s throughput is essentially only limited by the number of available technicians. The remaining bottleneck to the employment of either technique for tertiary evaluation of TRI is therefore mosquito processing [42]. A reliable method of high throughput mosquito homogenization involving minimal equipment costs will further enhance the scalability of the assays described here.

## Additional file

10.1186/s12936-015-0954-2 ECL-SB integrated optical density values for mosquito homogenate bands exposed by x-ray. Mosquitoes processed in separate blots but belonging to the same groups, for which cut-offs are different from the majority of mosquitoes in the same groups, are shown in this figure. All other details are as for Figure 1.
