# Supplementary material for: A comparison of Plasmodium falciparum circumsporozoite protein-based slot blot and ELISA immuno-assays for oocyst detection in mosquito homogenates
Source: Malar J. 2015 Nov 14;14:451. doi: 10.1186/s12936-015-0954-2 (PMC4647817; doi:10.1186/s12936-015-0954-2)
Supplement: Supplementary file 1 — 10.1186/s12936-015-0954-2 ECL-SB integrated optical density values for mosquito homogenate bands exposed by x-ray. Mosquitoes processed in separate blots but belonging to the same groups, for which cut-offs are different from the majority of mosquitoes in the same groups, are shown in this figure. All other details are as for Figure 1. [file 12936_2015_954_MOESM1_ESM.docx]

**A comparison of *Plasmodium falciparum* Circumsporozoite protein-based slot blot and ELISA immuno-assays for oocyst detection in mosquito homogenates**

Will Stone^1^, Bryan Grabias^2^, Kjerstin Lanke^1^, Emily Locke^3^, Diadier Diallo^3^, Ashley Birkett^3^, Merribeth Morin^3^, Teun Bousema^1,4^, Sanjai Kumar^2^

^1^ Department of Medical Microbiology, Radboud University Nijmegen Medical Centre, Nijmegen, The Netherlands

^2^ Laboratory of Emerging Pathogens, Division of Emerging and Transfusion Transmitted Diseases, Office of Blood Research and Review, Center for Biologics Evaluation and Research, Food and Drug Administration, Rockville, MD, USA

^3^ PATH Malaria Vaccine Initiative, Washington DC, USA

^4^ Department of Immunology and Infection, London School of Hygiene and Tropical Medicine, Keppel Street, London, UK

**Supplementary figures**

**
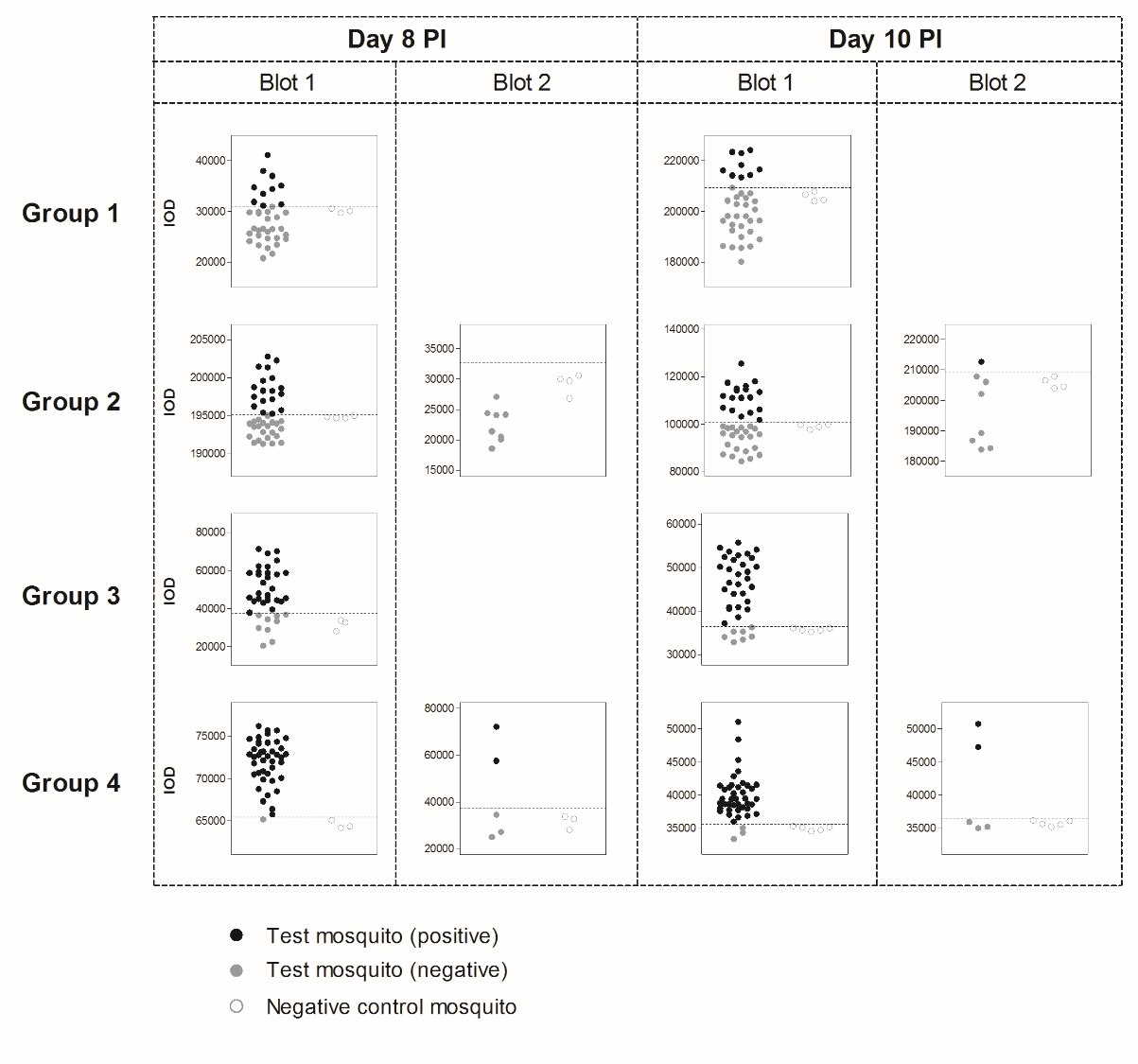
**

**Supplemental figure S1. ECL-SB integrated optical density values for mosquito homogenate bands exposed by x-ray.** Mosquitoes processed in separate blots but belonging to the same groups, for which cut-offs are different from the majority of mosquitoes in the same groups, are shown in this figure. All other details are as for *Figure 1*.
